# Circulating microRNAs targeting coagulation and fibrinolysis in patients with severe COVID-19

**DOI:** 10.1186/s12959-024-00649-w

**Published:** 2024-09-05

**Authors:** Tuukka A. Helin, Marja Lemponen, Katariina Immonen, Päivi Lakkisto, Lotta Joutsi-Korhonen

**Affiliations:** 1 HUS Diagnostic Center, Department of Clinical Chemistry, University of Helsinki and Helsinki University Hospital, POB 720, Helsinki, 00029 Finland; 2 Minerva Foundation Institute for Medical Research, Helsinki, Finland

**Keywords:** Blood coagulation, Blood platelets, COVID-19, Fibrinolysis, Inflammation, MicroRNAs

## Abstract

**Background:**

Coronavirus-19 disease (COVID-19) frequently causes coagulation disturbances. Data remains limited on the effects of microRNAs (miRNAs) on coagulation during COVID-19 infection. We aimed to analyze the comprehensive miRNA profile as well as coagulation markers and blood count in hospitalized COVID-19 patients.

**Methods:**

Citrated plasma samples from 40 patients (24 men and 16 women) hospitalized for COVID-19 were analyzed. Basic coagulation tests, von Willebrand factor (VWF), ADAMTS13, blood count, C-reactive protein, and 27 miRNAs known to associate with thrombosis or platelet activation were analyzed. MiRNAs were analyzed using quantitative reverse transcription polymerase chain reaction (RT qPCR), with 10 healthy controls serving as a comparator.

**Results:**

Among the patients, 15/36 (41%) had platelet count of over 360 × 10^9^/L and 10/36 (28%) had low hemoglobin of < 100 g/L, while 26/37 (72%) had high VWF of over 200 IU/dL. Patients had higher levels of the miRNAs miR-27b-3p, miR-320a-3p, miR-320b-3p, and miR-424-5p, whereas levels of miR-103a-3p and miR-145-5p were lower than those in healthy controls. In total, 11 miRNAs were associated with platelet count. Let-7b-3p was associated with low hemoglobin levels of < 100 g/L. miR-24-3p, miR-27b-3p, miR-126-3p, miR-145-5p and miR-338-5p associated with high VWF.

**Conclusion:**

COVID-19 patients differentially express miRNAs with target genes involved in fibrinolysis inhibition, coagulation activity, and increased inflammatory response. These findings support the notion that COVID-19 widely affects hemostasis, including platelets, coagulation and fibrinolysis.

**Supplementary Information:**

The online version contains supplementary material available at 10.1186/s12959-024-00649-w.

## Background

MicroRNAs (miRNAs) are key regulators of gene expression. They regulate messenger RNA (mRNA) function, inhibiting protein synthesis [1]. MiRNAs exert their effects dynamically, with rapid synthesis and varying half-lives. Using modern PCR methods, large panels of miRNAs can be examined in parallel in different clinical settings. Characteristic miRNA signatures can be found in arterial as well as venous thrombosis [2, 3]. Total circulating miRNA levels are generally upregulated in severe inflammatory conditions, such as bacterial sepsis [4]. In addition, miRNA markers have been found to associate with the presence and severity of COVID-19 infection [5]. Several miRNAs have been shown to be altered in bronchoalveolar fluid and plasma samples from COVID-19 patients [6, 7].

D-dimer is a fibrinogen degradation product that is increased in circulating blood when both coagulation activity and fibrinolysis are enhanced. Coagulation is frequently altered in COVID-19 patients, and a high D-dimer level as a sign of increased coagulation and fibrinolysis associates with poorer prognosis at hospital admission [8]. Our recent overview of coagulation laboratory tests showed anemia and increased coagulation activity in patients with COVID-19 [9]. In addition to disturbances in coagulation, enhanced platelet and von Willebrand factor (VWF) activation have been shown in COVID-19 [10]. ADAMTS13, a Disintegrin And Metalloproteinase with A Thrombospondin Motif 13, cleaves VWF multimers, and low levels have been shown in COVID-19 as well as other inflammatory conditions [11]. The relatively unique coagulation disturbance in COVID-19 infection highlights the need to comprehensively assess these patients. Not only does the data further our understanding of COVID-19 associated coagulopathy, but the findings may also be generalizable to other inflammatory processes.

The platelet contribution is critical for hemostasis. Especially during inflammation, platelet activation can significantly enhance thrombosis by upregulating tissue factor expression and initiating the formation of neutrophil extracellular traps [12, 13]. While lacking a nucleus, platelets continue to express mRNA and synthesize proteins and are an important source of miRNAs in circulation [14]. In the setting of infection, platelets and megakaryocytes are also part of the innate immune system, interacting with toll-like receptors, lectin receptors and the complement cascade. [15].

The aim of this study was to compile a comprehensive profile of both miRNAs and coagulation markers in COVID-19 patients treated during the early phases of the pandemic.

## Methods

### Patients

This was a retrospective, observational study using surplus plasma samples from COVID-19 patients. Plasma samples from 40 hospitalized COVID-19 patients (24 men and 16 women), median age 51 years, range 17–81, were collected during the COVID-19 pandemic in Finland, from May to September 2020. Blood count and C-reactive protein (CRP) levels were analyzed as part of routine care of the patient immediately, and the data were retrospectively accessed from the laboratory information system (LIS). Surplus citrated plasma samples obtained after routine coagulation tests were done were used for analyses. Whenever coagulation tests were requested from hospitalized COVID-19 patients, the first sample was included in our study. The samples and data were collected anonymously, and no further clinical details in addition to age and sex were available. The study was conducted with institutional approval (HUS/157/2020 and HUS/211/2020). Citrated plasma from these patients was centrifuged at 2500 g 15 min and frozen at -80 C. Coagulation tests and miRNA analyses were performed in batches from these samples. For miRNA analyses control samples from 10 healthy volunteers (5 women, 5 men) were processed in the same way as patient samples. Thus, all the samples underwent one thawing-refreezing cycle before miRNAs were analyzed, considering that it is known to alter miRNA profile [16]. Due to the small size of control group, we did not do age or sex-matching. However, neither patient nor control sex had any effect on the miRNA results (Mann Whitney U for difference, *p* > 0.05).

Of the 40 samples, two were excluded from the analysis because the plasma volume was too low for RNA isolation (< 200 ul). One sample was excluded due to hemolysis, which was assessed by visual inspection and spectrophotometry. The excluded sample had faint pink discoloration and a high 414 nm absorbance value. In addition, one sample was excluded from analysis because of high UniSp6 and Cel-miR-39 Cycle Threshold (CT) values in miRNA analysis, suggesting a possible inhibitor in the sample. Thus, 36 patient samples (21 men and 15 women) median age 51 years, range 17–81, were analyzed using a full set of data.

### MiRNA selection and analyses

To offer comprehensive view of miRNAs in COVID-19 patients, we selected miRNAs associated with primary hemostasis (platelets and VWF), as well as those associated with coagulation (association with thrombosis). We selected miRNAs highlighted in comprehensive reviews in literature [2, 3, 17]. A panel of 27 miRNAs were selected, which have been described in the literature to be associated with either venous or arterial thrombosis, being expressed abundantly in platelets, or having their effects on the VWF or ADAMTS13 gene (Table 1) [2, 3, 17].

**Table 1:**
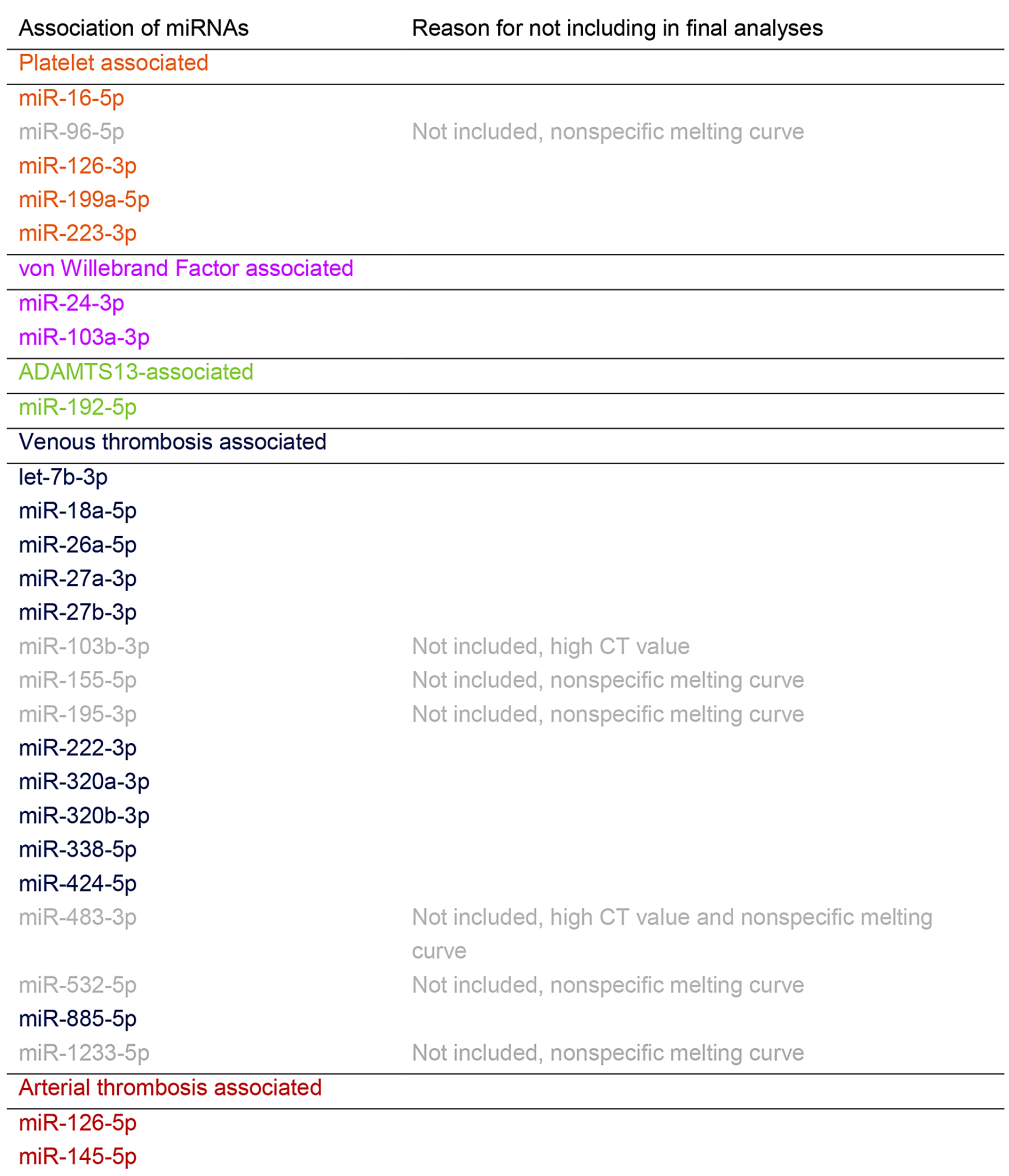
Twenty-seven miRNAs selected for the study based on the literature [2, 3, 17]. Twenty miRNAs were included in the final analyses

Before RNA isolation, plasma samples were analyzed for hemolysis by visual inspection and using NanoDrop One spectrophotometer (Thermo Fisher Scientific, Madison, Wisconsin, USA). The degree of hemolysis was assessed by measuring the oxyhemoglobin absorbance at a wavelength of 414 nm with three technical replicates performed for each sample.

Total RNA was isolated from 200 ul of plasma using a miRNeasy Serum/Plasma Advanced Kit (Qiagen, Hilden, Germany) according to manufacturer’s instructions. Spike-in synthetic Caenorhabditis elegans miRNA (Cel-miR-39) (Qiagen) was added as an external normalization control during RNA isolation and MS2 carrier RNA (Roche Diagnostics, Mannheim, Germany) to improve RNA yield. On-column DNase treatment was used to remove the potential genomic DNA contamination. RNA was eluted into 20 ul of nuclease-free water and stored at -80 C.

Reverse transcription (RT) was performed using the miRCURY LNA RT Kit (Qiagen). Spike-in UniSp6 (Qiagen) was added to control the RT reaction. As COVID-19 patients had been treated with heparin, a known inhibitor of miRNA quantification, heparinase treatment with 12U *Bacteroides* heparinase I (New England BioLabs, Ipswich, Massachusetts, USA) was performed during the RT reaction as described previously [18]. The RT reaction was incubated at 42 °C for 1 h, after which the reverse transcriptase was inactivated for 5 min at 95 °C.

The quantification of miRNAs was performed using the miRCURY LNA miRNA SYBR Green PCR kit (Qiagen) and miRNA-specific miRCURY LNA miRNA PCR Assay sets (Qiagen) on a LightCycler 480 (Roche Diagnostics). cDNA products were amplified for 45 cycles, with 10 s denaturation at 95 °C and 1 min annealing at 56 °C. The melting curve acquisition was carried out immediately after amplification. Seven miRNAs, miR-96-5p, miR-103b-3p, miR-155-5p, miR-195-3p, miR-483-3p, miR-532-5p, and miR-1233-5p were excluded from further analysis because of high CT values of over 35 and/or nonspecific melting curves. Expression values of target miRNAs were normalized to Cel-miR-39 as described earlier [19]. The mean CT values were 30.11 ± 1.50 for miR-192, 32.96 ± 1.14 for miR-338 and 31.27 ± 1.00 for let-7b. The mean CT values of all other studied miRNAs were below 30.

## Coagulation tests

Coagulation tests for prothrombin time (PT), activated partial thromboplastin time (APTT), thrombin time (TT), fibrinogen, coagulation factor VIII (FVIII) activity, antithrombin (HemosIL reagents), anti-Xa activity and D-Dimer (HemosIL D dimer HS 500, FEU units), VWF activity and antigen as well as blood count, inflammatory marker CRP and creatinine were performed using routine methods. The following instruments were used: coagulation with ACL TOP^®^ 500 and 750 analyzers, (Instrumentation Laboratory, Naples, Italy); blood count with Sysmex^®^ XN-9000 (Kobe, Japan); CRP and creatinine with Siemens Atellica^®^ Solution (Siemens Healthineers, München, Germany). These have been previously described in a larger cohort [9]. In addition, here, we performed von VWF GPIb activity assay and antigen with the ACL TOP^®^ 500/750, with HemosIL reagents. We also performed chemiluminescent ADAMTS13 assay and VWF collagen binding assay (both with Acustar^®^ analyzer, Werfen, Barcelona, Spain). The reported reference ranges for each variable (Table 2) are used in our accredited laboratory.

### Statistics

For all variables, median, range, and interquartile range were calculated. Spearman correlation and Mann-Whitney U test were used when appropriate. The analyses were performed using R statistics software [20].

## Results

### Coagulation and inflammatory markers in COVID-19 patients

The results of the coagulation tests, blood count, and CRP analysis on the COVID-19 patients are presented in Table 2. The median fibrinogen, D-dimer, FVIII and VWF levels were all above the reference interval, and median platelet count was high normal (330 × 10^9^/L). Among the patients, 15/36 (41%) had thrombocytosis and 10/36 (28%) had hemoglobin concentration less than 100 g/L, while 26/37 (72%) had WF of over 200 IU/dL. In addition, 18/36 (50%) patients had high D-dimer levels of over 1.5 mg/L. Due to the nature of our study, we do not have laboratory results from the patients prior to the study. In our previous study, hemoglobin trended lower and fibrinogen and D-dimer higher after positive SARS-CoV-2 PCR test, in hospitalized COVID-19 patients [9].

**Table 2:** Summary of laboratory test results in the 36 patient samples. Variables with medians outside the reference intervals are marked in bold text

	Median	Range	Interquartile range	NAs	Reference range
*Coagulation tests*					
**D-dimer (mg/L)**	**1.4**	**0.2–23.9**	**0.8–3.4**		**< 0.5**
**VWF: Act (IU/dL)**	**279**	**71–816**	**197–327**	**1**	**50–190**
**VWF: Ag (IU/dL)**	**377**	**118–1202**	**283–461**	**1**	**50–190**
**VWF: CB (%)**	**224**	**7-579**	**164–289**	**4**	**55–180**
**Fibrinogen (g/L)**	**5.9**	**1.8–9.2**	**5-7.3**		**2.0–4.0**
**FVIII activity (IU/mL)**	**216**	**91–461**	**168–280**		**60–160**
**Anti-Xa (IU/mL)**	**0.15**	**0-0.52**	**0.04–0.27**	**1**	**< 0.1**
ADAMTS13 (%)	71	9-123	58–90	1	60–130
PT (%)	94	60–171	86–101		70–130
APTT (s)	32	17–106	29–35		28–37
Thrombin time (s)	20	17–28	18–21	4	17–25
Antithrombin (IU/dL)	89	20–121	79–102		85–125
Hematology					
Hemoglobin (g/L)	114	79–148	100–129		^*^
Leukocytes (x10^9^/L)	7.0	2.3–31.5	5.3–10.6		3.4–8.2
Platelet count (x10^9^/L)	330	42–585	232–415		150–360
Chemistry					
**CRP (mg/L)**	**61**	**3-266**	**30–110**		**< 4**
Creatinine (µmol/L)	48	28–279	41–67		^**^

* Hemoglobin ref interval 134–167 g/L for men, 117–155 g/L for women. ** Creatinine ref interval 50–90 µmol/L for women, 60–100 µmol/L for men

APTT, activated partial thromboplastin time; CRP, C reactive protein; PT, prothrombin time; VWF: Act, von Willebrand factor GPIb activity; VWF: Ag, VWF antigen VWF: CB, von Willebrand factor, collagen binding assay; NAs, samples with no results

### miRNA profile in COVID-19 patients compared with healthy controls

**Fig. 1:**
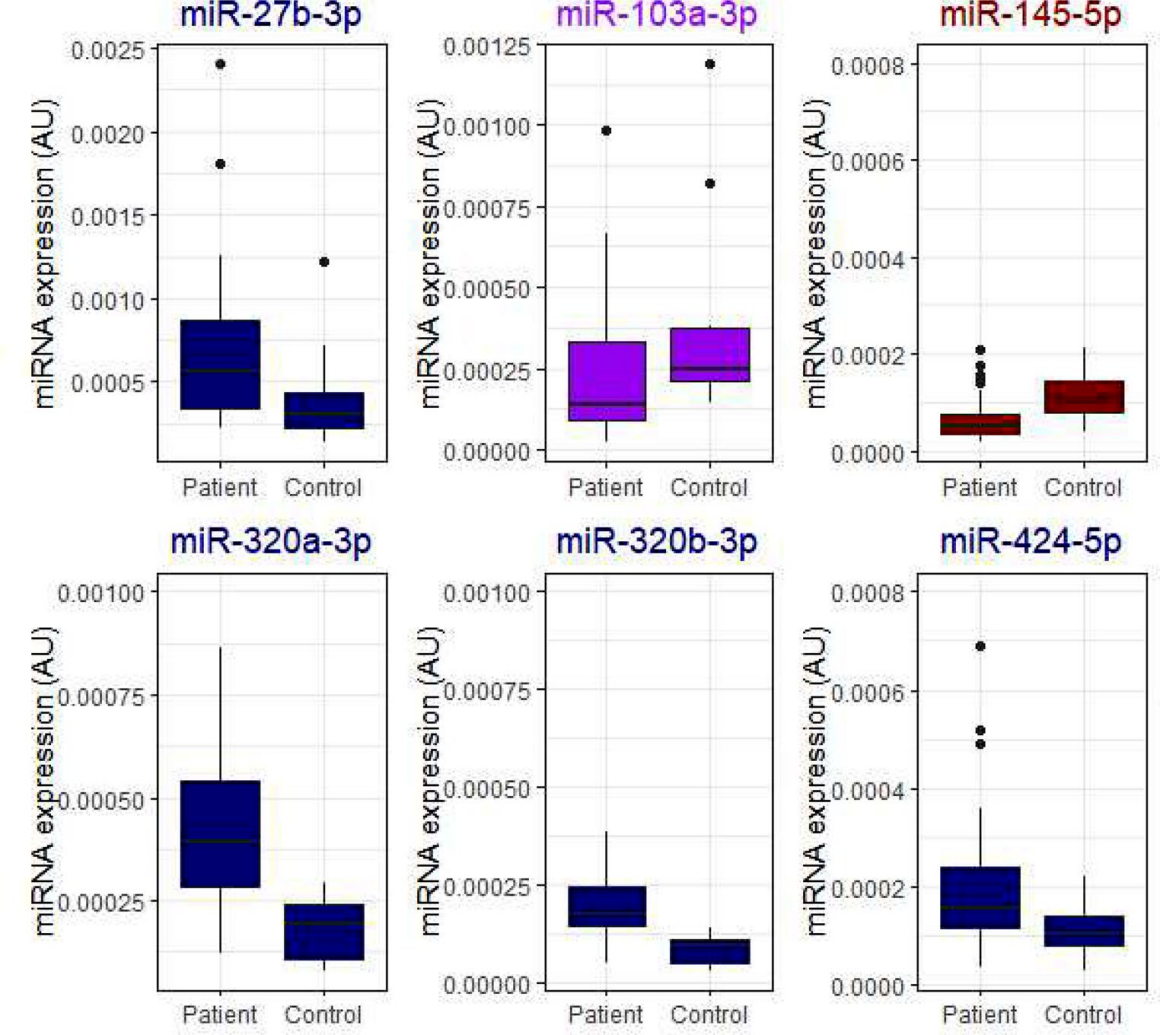
COVID-19 patients had higher levels of miRNAs miR-27b-3p, miR-320a-3p, miR-320b-3p and miR-424-5p, while levels of miR-103a-3p and miR-145-5p were lower, although with miR-103a-3p the top quartiles overlap. Mann-Whitey U for difference *p* < 0.05. For miR-320a/b, the difference was highly significant, *p* < 0.001. For comparisons, MiRNAs in literature associated with venous thrombosis are marked in navy, those associated with von Willebrand -factor are marked in purple and those associated with arterial thrombosis are marked in crimson. Data are presented as boxplots with median (thick line), 25th and 75th percentile distribution (box limits), range (whiskers) and outliers (points). AU, arbitrary units

MiR-27b-3p, miR-320a/b-3p, and miR-424-5p were higher and miR-103a-3p and miR-145-5p were lower in COVID-19 patients compared with controls, although in miR-103a-3p the top quartiles overlap (Fig. 1; Mann Whitney U for difference, *p* < 0.05).

### MiRNA associations with blood count, coagulation, and inflammation markers

**Fig. 2:**
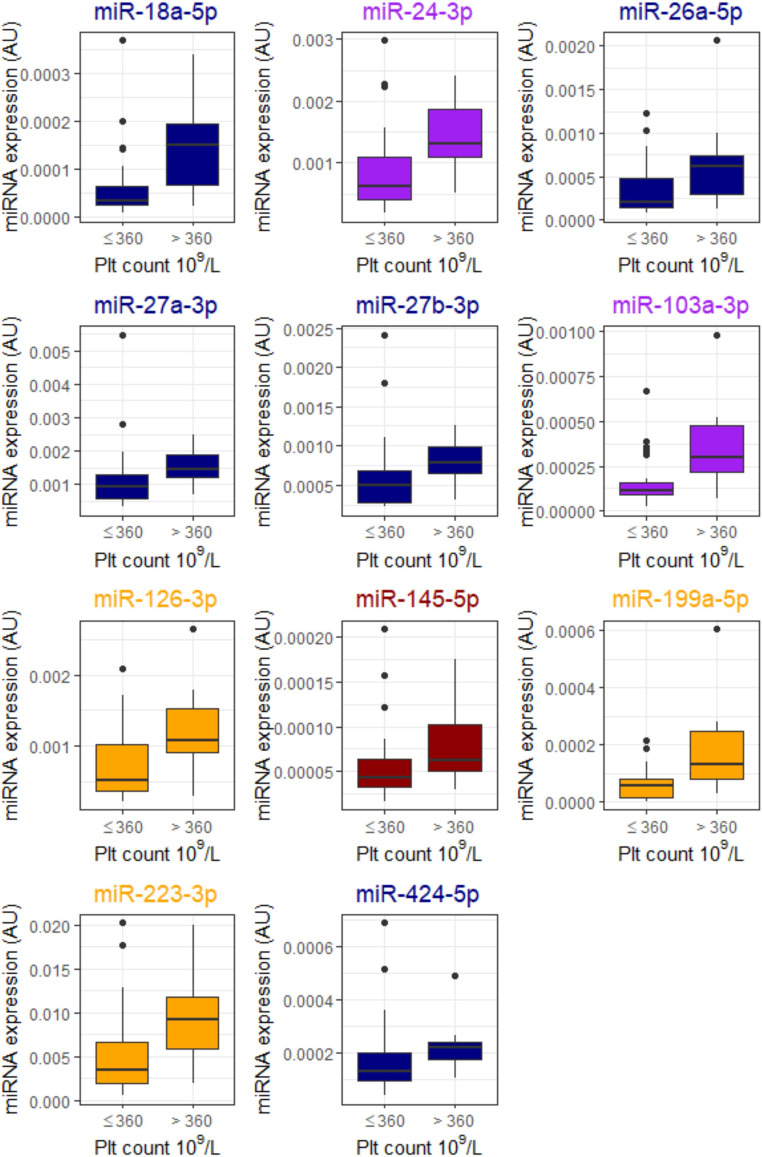
Samples with high platelet count (over 360 × 10^9^/L), had higher expression of 11 miRNAs. Association of miRNAs with platelet count. Mann-Whitey U for difference *p* < 0.05. For comparisons, MiRNAs in the literature associated with latelets are marked in orange, venous thrombosis -associated miRNAs in navy, von Willebrand-factor-associated miRNAs in purple, and arterial thrombosis -associated miRNAs in crimson. Data are presented as boxplots with median (thick line), 25th and 75th percentile distribution (box limits), range (whiskers) and outliers (points). AU, arbitrary units

**Fig. 3:**
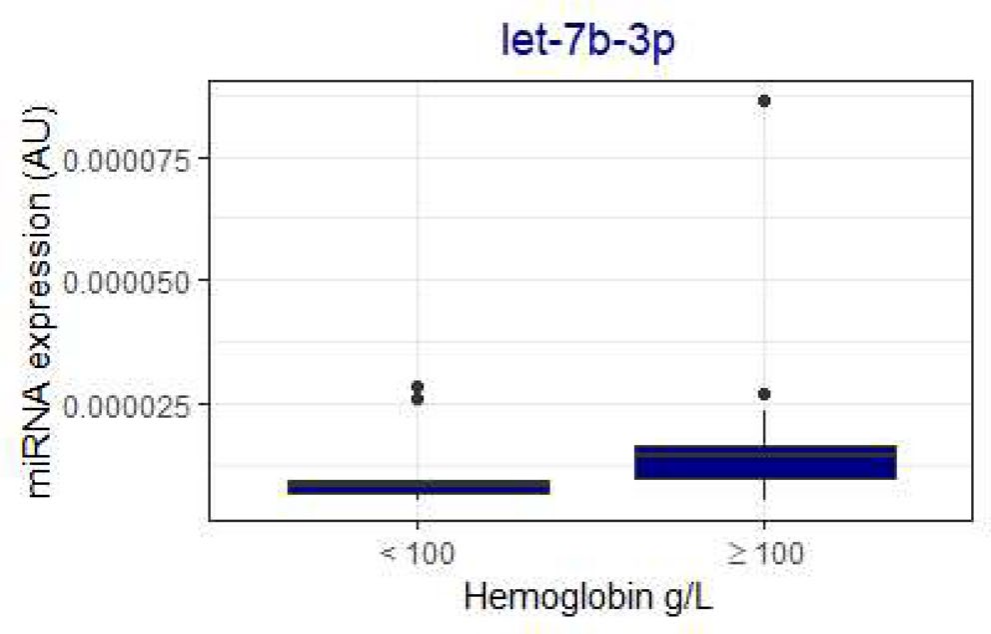
Low hemoglobin levels (under 100 g/L) were associated with lower let-7b-3p expression. Mann-Whitney U for difference < 0.05. The association was independent of platelet count. In the literature, let-7b-3p is associated with venous thromboembolism and is marked in navy. Data are presented as boxplots with median (thick line), 25th and 75th percentile distribution (box limits), range (whiskers) and outliers (points). AU, arbitrary units

**Fig. 4:**
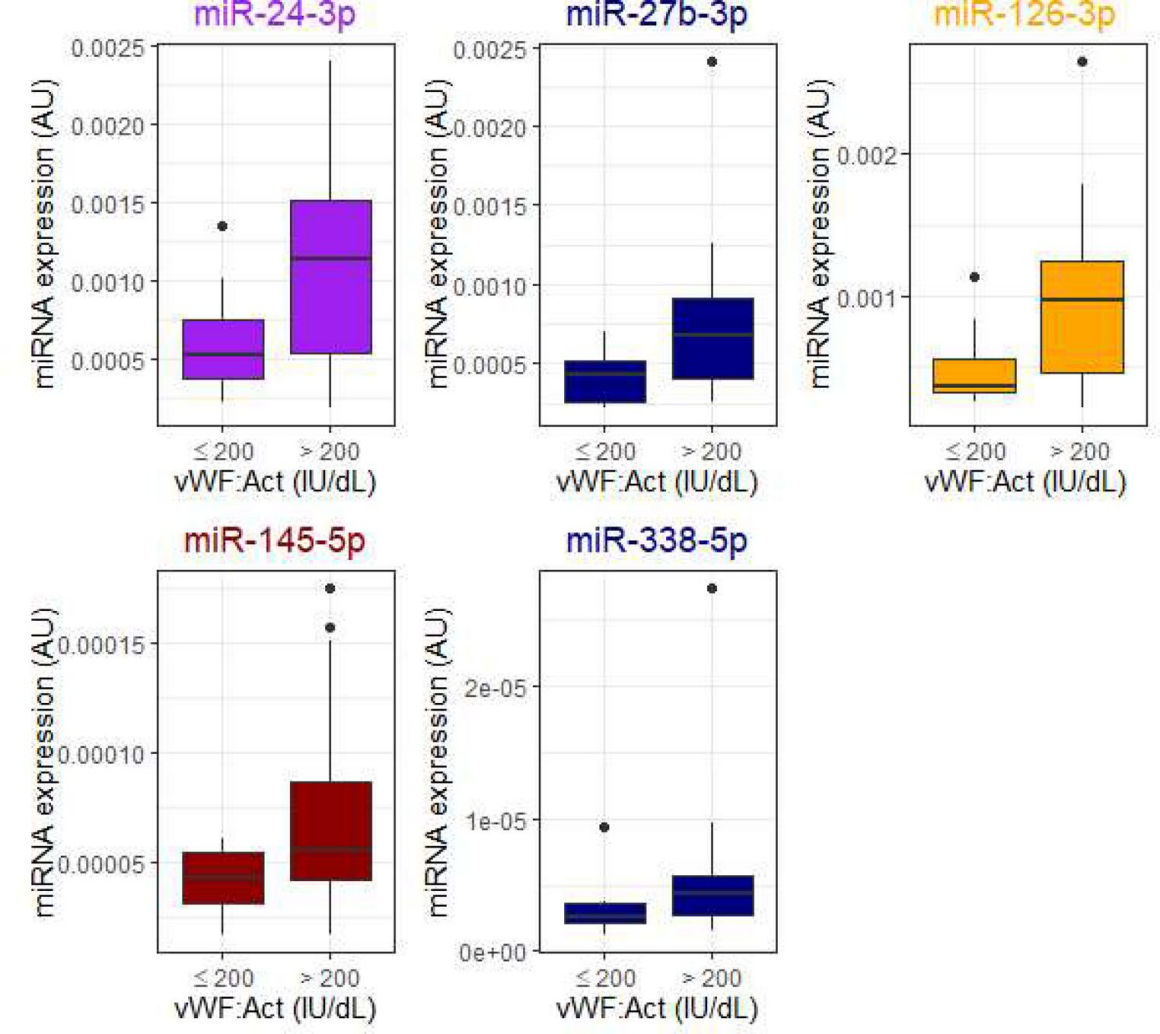
Von Willebrand factor activity (vWF Act) level associated with increased concentrations of miR-24-3p, miR-27b-3p, miR-126-3p, miR-145-5p, and miR-338-5p. Mann-Whitney U for difference < 0.05 For comparisons, miRNAs in the literature associated with platelets are marked in orange, those associated with venous thrombosis in navy, those associated with von Willebrand-factor in purple, and those associated with arterial thrombosis in crimson. Data are presented as boxplots with median (thick line), 25th and 75th percentile distribution (box limits), range (whiskers) and outliers (points). AU, arbitrary units

Several miRNAs were associated with platelet count, with 11 achieving statistical significance (Mann-Whitney U *p* < 0.05) in comparisons (Fig. 2). Let-7b-3p was associated with low hemoglobin levels of under 100 g/L (Fig. 3) and was not associated with platelet count. Five miRNAs, miR-24-3p, miR-27b-3p, miR-126-3p, miR-145-5p, and miR-338-5p, were higher in patients with high VWF levels of over 200 IU/dL (Fig. 4). D-dimer level did not associate with miRNA expression in these patients.

**Fig. 5:**
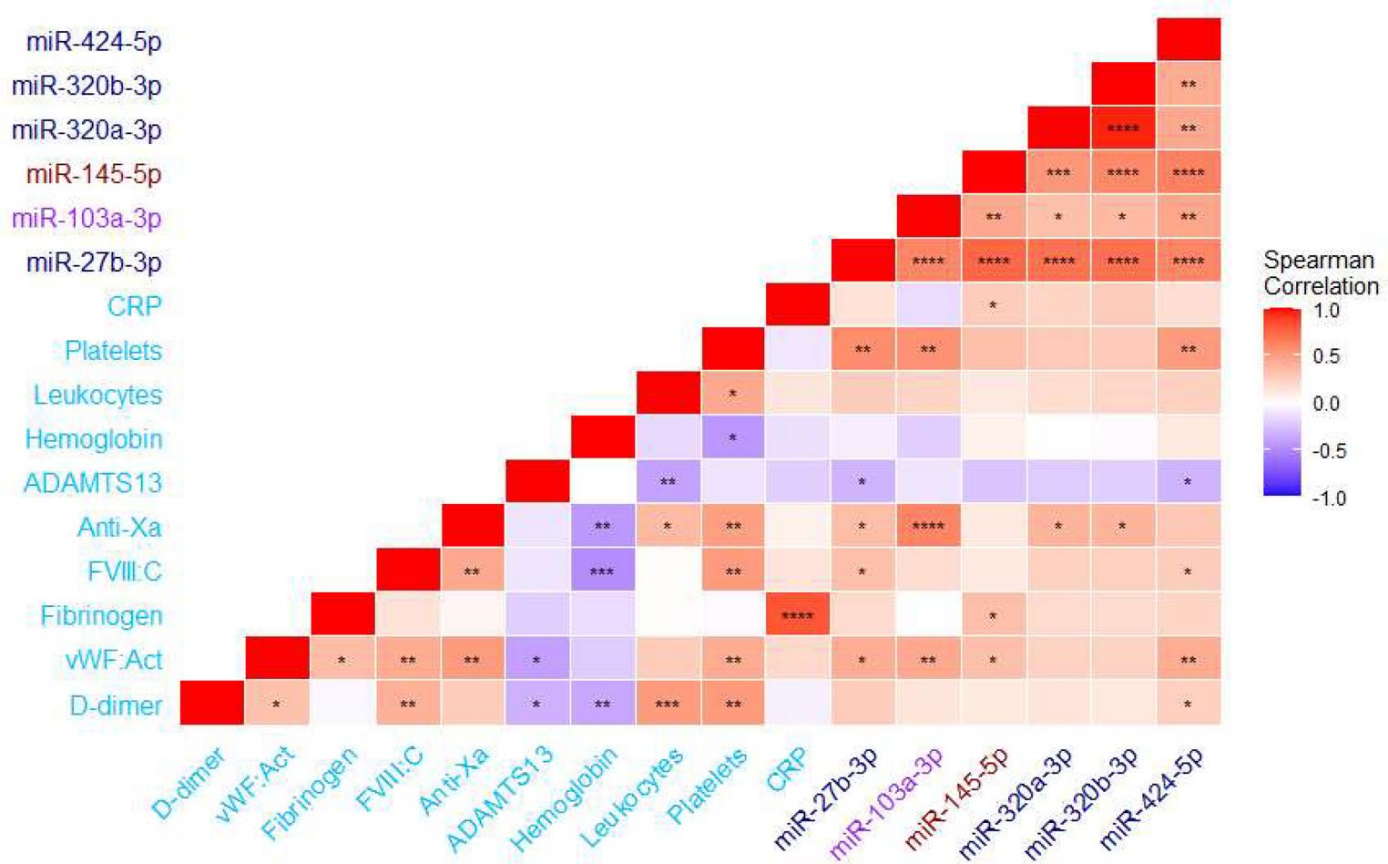
Correlation matrix of miRNAs significantly different between COVID-19 patients and controls, with coagulation and blood count variables. Significant correlations (*p* < 0.05) are marked with * *p* < 0.05, ** *p* < 0.01, *** *p* < 0.001, ****p < 0.0001. For comparisons, miRNAs associated in the literature with venous thrombosis are marked in navy, those with von Willebrand-factor in purple, and those with arterial thrombosis in crimson. Coagulation, inflammation, and blood count variables are marked in sky-blue

Spearman correlations of miRNAs differing between patients and controls are shown in Fig. 5, while correlations of all the studied miRNAs are shown in the Supplementary Figure. MiRNAs were highly correlated with one another (*p* < 0.001), except for miR-192-5p and miR-885-5p. MiR-103a-3p level was lower in COVID-19 patients than in controls, and it strongly correlated with anti-Xa and platelet count (Fig. 5). In the miRNAs with differing levels in COVID-19 patients and controls, miR-145-5p correlated with both CRP and fibrinogen (*p* < 0.05). In these miRNAs, D-dimer only correlated with miR-424-5p, whereas ADAMTS13 had a negative correlation with miR-27b-3p and miR-424-5p (Fig. 5).

## Discussion

In previous literature, COVID-19 has been shown to widely impact coagulation [8–11]. COVID-19 has also been shown to impact many miRNAs, including coagulation associated miR-27b-3p, miR-145-5p, and mir-320a/b [7, 21, 22]. Different miRNA signatures have been shown in critically ill COVID-19 patients as opposed to hospital-ward-treated patients, including altered miR-16-5p and miR-192-5p expression, which have been shown to associate with platelets and ADAMTS13, respectively [2, 3, 17, 23]. In addition, many miRNAs that are dysregulated in COVID-19 have been proposed to contribute to the development of lung fibrosis [24] However, limited data are available on the extent of miRNA associations with coagulation in the setting of COVID-19, which we set out to study. Here, MiR-27b-3p, miR-320a/b-3p, and miR-424-5p were higher in COVID-19 patients and miR-103a-3p and miR-145-5p were lower in COVID-19 patients compared with controls (Fig. 1). In previous literature, miR-103a-3p has known targets with VWF, miR-145-5p is associated with arterial thrombosis and smooth muscle proliferation, as well as ADAMTS13 levels, and miR-27b-3p, miR-320a/b-3p, and miR-424-5p are associated with venous thromboembolism [3]. miRNA genes and pathways were explored with the connections to thrombosis and COVID-19 using the miRwalk and DianaTools databases [25, 26], and their cell expression using Patil et al. microRNAome data [27].

miR-27b -3p is part of the miR-23b-27b-24-1 cluster located on chromosome 9. miR-27b-3p has been implicated in non-alcoholic fatty liver disease and is overexpressed in mice during inflammatory conditions [28]. It targets plasminogen and plasminogen activator genes (PLAU and PLG) [25]. Plasminogen is a key activator of fibrinolysis, suggesting a miR-27b-3p inhibition as a possible mechanism for thrombosis development. miR-27b-3p is highly expressed in various cell types, including vascular smooth muscle cells and fibroblasts [27]. In a recent study, miR-27b-3p was shown to be higher in severe, ICU treated COVID-19 patients than ward-treated patients [7] The contribution of miR-27b-3p to thrombosis is not clear-cut, however, lower levels of the miRNA have previously been implicated in recurrent venous thromboembolism [17, 29]. In our study, it negatively correlated with ADAMTS13, and low ADAMTS13 levels have been shown to be associated with a more severe COVID-19 phenotype (Fig. 5) [30].

MiR-103a-3p targets SERPINE 1 (coding protein plasminogen activator inhibitor, PAI-1) and TNF genes [25] In our study, miR-103a-3p levels were lower in COVID-19 patients compared with controls, although the top quartiles overlapped with controls (Fig. 1). This suggests higher levels of PAI-1 in at least some of COVID-19 patients compared to controls (less inhibitory miRNA effect). This would contribute to lower fibrinolysis and increased thrombin formation. Indeed, in a study with 113 hospitalized COVID-19 patients, increased concentrations of PAI-1 and attenuated clot lysis were observed [31]. MiR-103a-3p is highly expressed in various cell types, with inflammatory B-lymphocytes exhibiting the highest expression [27]. MiR-103a-3p strongly correlated with anti-Xa activity, suggesting that heparin antithrombotic and anti-inflammatory effects might modulate miRNA expression in these COVID-19 patients, with increased expressions in heparinized patients (Fig. 5).

MiR-145-5p levels were also lower in COVID-19 patients than in controls (Fig. 1). MiR-145-5p also targets SERPINE1, as well as tissue factor and IL6 genes, suggesting that tissue factor activation might have been higher in COVID-19 patients [25]. Yet, in our study, MiR-145-5p was also associated with high platelet counts (over 360 × 10^9^/L) and high VWF levels (over 200 IU/dL; Figs. 2 and 4), findings commonly occurring in more severe COVID-19 infection. MiR-145-5p is most highly expressed in fibroblasts, but not in platelets or endothelium [27]. However, platelets contribute to fibroblast growth and fibroblasts also express proteins that promote coagulation, highlighting the role of miR-145-5p in this interplay [32]. In a previous study, COVID-19 patients exhibited lower amounts of exosomal miR-145-5p, in concordance with our findings here [21] However, in two other studies, miR-145-5p levels were higher in serum or whole blood of hospitalized COVID-19 patients, respectively. [33, 34] Differences in sample type or RNA extraction protocols might explain the differences to our study. Patient serum or PaxGene Blood RNA System whole blood samples were used in those studies, whereas we used Na-citrate plasma samples. Lower miR-145-5p concentrations are seen in conjunction with arterial thrombosis, during inflammatory conditions, such as atherosclerosis and diabetes, and increased tissue factor activation in animal models [17, 35, 36]. Our finding of lower miR-145-5p levels thus concords with the inflammatory response associated with COVID-19 infection. The distinction however, is not clear, as patients with high platelet counts and VWF had higher expression.

In our study, miR-320a-3p and miR-320b-3p levels were higher in COVID-19 patients than in controls (Fig. 1). MiR-320a/b-3p target cytokine signaling, the thromboxane gene, and the CRP gene [25]. In contrast, thromboxane activation might be stimulated by miR-320a/b-3p activation, with corresponding higher primary hemostasis activation [37]. MiR-320a/b-3p are present in high concentrations in platelets, regulate endothelial adhesion, and high levels are associated with type 2 diabetes, as well as venous thromboembolism [17, 27, 38–40]. Our findings are in contrast to a previous study, where miR-320a/b-3p were downregulated in severe COVID-19 patients [22] In that study, PaxGene Blood RNA System was used for whole blood collection, while we collected routine Na-citrate plasma samples. Contrary data also exists, with two studies reporting miR-320a-3p upregulation in COVID-19 [41, 42]. Differences in sample type and RNA extraction protocols might explain the difference in miR320a/b-3p levels [43]. Our finding also concords with our previous study, where platelet-associated extracellular vesicles were higher in COVID-19 patients than controls [44]. Here, high VWF, a key primary hemostasis activator, also associated with high miR-338-5p (Fig. 4). As miR-338-5p targets the thromboxane gene and fibroblast growth receptor signaling and has been described to associate with venous thrombosis [17], our findings are novel and interesting.

MiR-424-5p targets many pathways, including p53 signaling, TGF-beta signaling, and fatty acid biosynthesis, and its regulation is involved in several cancers [26] It also has a target site in the interleukin 1 gene [25]. It has been shown to be elevated in plasma in patients who have developed thrombosis with concomitant high D-dimer [45]. In computational analysis, miR-424-5p exhibited a probable direct interaction with coronavirus, and it has been shown to be elevated in COVID-19 patients both in the acute setting and during hospitalization [33, 46, 47]. The high miR-424-5p levels probably reflect the high inflammation and possibly viral load of the patients.

In our study, low hemoglobin was only associated with let-7b-3p (Fig. 3). Let-7b-3p is downregulated in severely ill chronic thromboembolic pulmonary hypertension patients, and positively correlates with D-dimer and PAI-1 [48]. Let-7b-3p is highly expressed in various cell types, including endothelial cells [27]. The findings of low hemoglobin, high D-dimer, and high PAI-1 are all associated with more severe COVID-19 infection [9, 31], thus making our findings even more interesting. Let-7b-3p targets the angiopoietin-2 gene, and lower levels are associated with chronic thromboembolic pulmonary embolism [48].

The main limitations of this study include the relatively small size of the patient population, as well as the lack of complete clinical data on the patients. As we do not have pre-COVID-19 laboratory results or medical history of the patients, we are unable to make further comparisons or subgrouping of the patients. The pre-COVID-19 coagulation profile may already be altered in some individuals. Yet, in our previous study, we have shown clear coagulation disturbances in COVID-19 infection, strengthening the findings of this study [9]. Furthermore, we did not have a full set of blood count and coagulation panel on the control individuals, precluding comparison of miRNA levels to blood count in controls, although their results are likely within the reference intervals. A fourth limitation is sample handling, with refreezing and thawing probably accentuating the role of platelets, making firm conclusions of platelet-derived, platelet count-associated miRNAs difficult [16]. Indeed, 11 miRNAs were associated with platelet count (Fig. 2). On the other hand, platelets are key regulators of hemostasis, and our sample protocol highlights their role. The main strength of this study was the comprehensive assessment of both coagulation variables and a large miRNA panel. The findings highlight the role of platelets as a source of miRNAs, with many of the findings probably relating to increased platelet concentration in COVID-19 patients. This study also provides data on the preanalytical methodology and implications of miRNAs. EDTA plasma samples are commonly used in miRNA analyses; however, Na-citrate plasma is also suitable, as evidenced here [49]. Na-citrate may also cause lower artificial in vitro platelet activation than EDTA [50].

In conclusion, COVID-19 patients differentially express several miRNAs with known target genes in the activation of primary hemostasis and coagulation, inhibition of fibrinolysis, and activation of the inflammatory response. Our study highlights the role of miRNAs in the regulation of hemostasis and coagulation during COVID-19 infection. Further studies are required to identify prognostic markers related to outcomes. Larger patient cohorts, with matched controls are needed to enable subgrouping and to elucidate mechanism behind the miRNA changes observed here. Nevertheless, this study reveals new data on miRNAs in the interplay between inflammation and coagulation.

## Electronic supplementary material

Below is the link to the electronic supplementary material.

Supplementary Material 1

## Data Availability

The datasets used and analysed during the current study are available from the corresponding author on reasonable request.
