# Supplementary material for: Circulating microRNAs targeting coagulation and fibrinolysis in patients with severe COVID-19
Source: Thromb J. 2024 Sep 5;22:80. doi: 10.1186/s12959-024-00649-w (PMC11375984; doi:10.1186/s12959-024-00649-w)

Supplemental Figure: Spearman correlation matrix for all measured laboratory variables and miRNAs. Significant correlations (p<0.05) are marked with * p<0.05, ** p<0.01, *** p<0.001, ****p<0.0001.

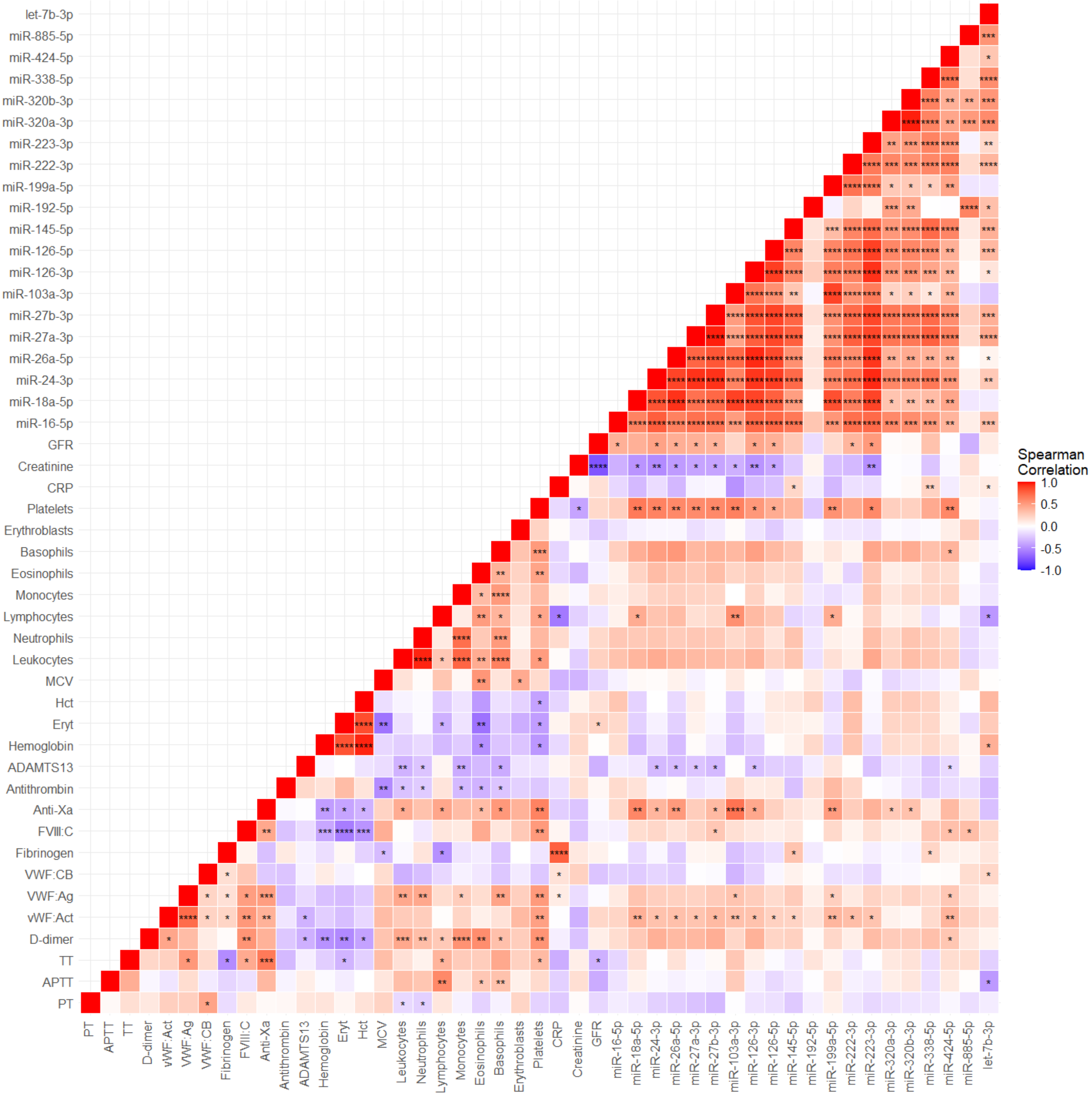

Supplement: Supplementary file 1 — Supplementary Material 1 [file 12959_2024_649_MOESM1_ESM.pdf]
